# Reply to the Letter on: Penile reconstruction in a newborn following complicated circumcision: A case report

**DOI:** 10.1016/j.ijscr.2019.02.004

**Published:** 2019-02-27

**Authors:** M.M. Al-Qattan

**Affiliations:** Division of Plastic Surgery, Department of Surgery, King Saud University, PO Box 18097, Riyadh, 11417, Saudi Arabia

Dear Editor

We appreciate the comments. Currently the erection in our patient is complete without restrictions. However, we are aware of the possibility of problems with growth. Hence, we have informed the parents regarding these possible problems in the future. Intra-operatively, we considered using scrotal flaps for the coverage of penile shaft. However. The diameter of the penile shaft was so small and the scrotal flap was thought to be too thick and hence would hide the definition of the shaft at this age. The excellent myo-cutaneous flap described by the authors still remains an option if needed in the future. We promise the readers to write a letter to the Editor in the future to document if such problems would arise.

## Conflict of interest

None.

## Funding

None.

## Ethical approval

The original study was approved by the research committee, National Hospital (Care), Riyadh, Saudi Arabia. The current reply does not require this.

## Consent

Not applicable

## Author contribution

Reply is from the senior author on behalf of all authors.

## Registration of research studies

NA.

## Guarantor

M M Al-Qattan.

